# Expression Profile of Laccase Gene Family in White-Rot Basidiomycete *Lentinula edodes* under Different Environmental Stresses

**DOI:** 10.3390/genes10121045

**Published:** 2019-12-16

**Authors:** Lianlian Yan, Ruiping Xu, Yinbing Bian, Hongxian Li, Yan Zhou

**Affiliations:** 1 Institute of Applied Mycology, College of Plant Science and Technology, Huazhong Agricultural University, Wuhan 430070, China; yylaputa@163.com (L.Y.); mushroomhzau@163.com (R.X.); bianyinbing@mail.hzau.edu.cn (Y.B.); 1628629313@163.com (H.L.); 2 Key Laboratory of Agro-Microbial Resource Comprehensive Utilization, Ministry of Agriculture, Huazhong Agricultural University, Wuhan 430070, China

**Keywords:** *Lentinula edodes*, laccase gene family, low temperature, high temperature, photoperiod, carbon sources

## Abstract

Laccases belong to ligninolytic enzymes and play important roles in various biological processes of filamentous fungi, including fruiting-body formation and lignin degradation. The process of fruiting-body development in *Lentinula edodes* is complex and is greatly affected by environmental conditions. In this paper, 14 multicopper oxidase-encoding (laccase) genes were analyzed in the draft genome sequence of *L. edodes* strain W1-26, followed by a search of multiple stress-related *Cis*-elements in the promoter region of these laccase genes, and then a transcription profile analysis of 14 laccase genes (*Lelcc*) under the conditions of different carbon sources, temperatures, and photoperiods. All laccase genes were significantly regulated by varying carbon source materials. The expression of only two laccase genes (*Lelcc5* and *Lelcc6*) was induced by sodium-lignosulphonate and the expression of most laccase genes was specifically upregulated in glucose medium. Under different temperature conditions, the expression levels of most laccase genes decreased at 39 °C and transcription was significantly increased for *Lelcc1*, *Lelcc4*, *Lelcc5*, *Lelcc9*, *Lelcc12*, *Lelcc13*, and *Lelcc14* after induction for 24 h at 10 °C, indicating their involvement in primordium differentiation. Tyrosinase, which is involved in melanin synthesis, was clustered with the same group as *Lelcc4* and *Lelcc7* in all the different photoperiod treatments. Meanwhile, five laccase genes (*Lelcc8*, *Lelcc9*, *Lelcc12*, *Lelcc13*, and *Lelcc14*) showed similar expression profiles to that of two blue light receptor genes (*LephrA* and *LephrB*) in the 12 h light/12 h dark treatment, suggesting the involvement of laccase genes in the adaptation process of *L. edodes* to the changing environment and fruiting-body formation. This study contributes to our understanding of the function of the different *Lelcc* genes and facilitates the screening of key genes from the laccase gene family for further functional research.

## 1. Introduction

Laccase (benzenediol: oxygen oxidoreductase, EC 1.10.3.2) is a group of phenoloxidases containing copper atoms in the catalytic center, and is also a member of the blue multicopper oxidase family (MCO) [1]. Laccases have been widely studied for their potential industrial applications in pulp bleaching, dye decolorization, detoxification of environmental pollutants, and revalorization of wastes and wastewaters [2,3,4]. Laccase was shown to contain three types of coppers (type 1, type 2, and type 3) [5,6,7]. A set of four ungapped sequence regions L1–L4 are used to distinguish laccases within the broader class of MCOs [6,7]. Laccases are also widely distributed in fungi, bacteria, and insects, but not plants [7]. Sequence comparison and transcription analysis of several fungi demonstrated that laccases were encoded by multigene families [8,9,10,11]. This gene redundancy suggests the differences in their physicochemical and regulatory mechanisms, especially their physiological roles in response to nutrition change and environmental stress [12,13]. The intricacy of laccase genes families in fungi cumbers the further study of these genes.

In plants, laccases participate in the radical-based mechanisms of lignin polymer formation [14,15], while in fungi, laccases are thought to play a variety of physiological roles, including stress defense, melanin synthesis [10], fruiting-body formation, and lignin degradation [7]. The transcription of laccase genes in various organisms (mostly fungi) is affected by many factors, such as the type and nature of nutrients, temperature, pH, and chemical inducers, and also varies with the phases of fruiting-body development [9,11,12,16]. In recent years, a number of reports were published regarding the genetic functional analysis of laccases in mushrooms, such as *Pleurotus ostreatus*, *Hypsizygus marmoreus*, and *Lentinula edodes* [17,18,19,20]. Comprehensive knowledge regarding the laccase gene family in terms of induced transcription under diverse conditions could provide an understanding of the functions of different laccase genes.

*L. edodes*, an important cultivated edible and medicinal mushroom, belongs to the wood-colonizing white-rot species. The formation process of its fruiting bodies is mediated by cellular processes and genetic, physiological, and environmental factors [21]. Specifically, light, low temperature, and substrates are the key factors that affect the induction of brown film (BF) formation and primordium initiation, with the expression of laccase genes significantly upregulated during the two stages [21,22,23]. The correlation analysis showed that the laccase activity in the vegetative growth stage was closely related to the time when *L. edodes* entered the reproductive stage. The greater the enzyme activity, the earlier the brown film formation and primordium initiation [24]. In-depth systematic exploration of how *L. edodes* adapts to various environmental conditions and starts the reproductive growth stage is particularly important for efficient cultivation of *L. edodes*.

Thus far, 14 laccase genes (*Lelcc*) have been identified in *L. edodes* [25]. The expression of the laccase genes was reported to be induced by different environmental factors in *L. edodes* and varied during fruiting-body development [11,21,23,26]. The genome data of *L. edodes* W1-26 were published by our laboratory [25]. Additionally, our analyses of the transcriptome and secretion proteome data indicated that laccases in *L. edodes* respond to stresses such as heat and carbon sources [27,28]. Therefore, it is necessary to further explore the response mechanisms of *L. edodes* to various environmental conditions to improve our understanding of its growth characteristics and enhance its cultivation efficiency.

The purpose of the present study was to analyze the expression profile of the laccase gene family in *L. edodes* under different environmental stresses, such as photoperiods, carbon sources, and temperature. The results could facilitate the understanding of the laccase function of white-rot fungi regarding their adaptive capacity to environment and provide a convenient way to select interesting genes from the *L. edodus* laccase family genes for further research.

## 2. Materials and Methods

### 2.1. Phylogenetic Analysis of Laccase Genes of L. edodes

The laccase sequences of *L. edodes* W1-26 (a single spore culture derived from the W1 strain) were downloaded from the *L. edodes* Genome Database http://legdb.chenlianfu.com/index.html [25]. Subsequently, the protein sequences of the identified members of *L. edodes* laccase gene family were analyzed with EXPASY PROTOPARAM4 to obtain the molecular weight and theoretical isoelectric point (pI). Signal peptides of each laccase were predicted using SignalP algorithm (http://www.cbs.dtu.dk/services/SignalP/). Four ungapped sequence regions (L1–L4) were used to distinguish between the laccases in *L. edodes*.

All 14 laccase genes were analyzed phylogenetically by alignment of the respective amino acid sequences using ClustalW software (http://www.ddbj.nig.ac.jp/search/clustalw-j.html). The phylogram was constructed using the neighbor-joining method, and trees were drawn using FigTree (http://tree.bio.ed.ac.uk/software/fgtree/). Bootstrapping was carried out with 1000 replications.

### 2.2. Promoter Analysis of Laccase Genes in L. edodes

The upstream regions (1.5 kb) of the laccase gene sequences were downloaded from the *L. edodes* Genome Database (http://legdb.chenlianfu.com/index.html) and used for search of *Cis*-elements online by Yeastract (Yeast Search for Transcriptional Regulators and Consensus Tracking, http://www.yeastract.com/index.php). The *Cis*-elements predicted by Yeastract were classed to four main groups according to their putative function (Supplementary Table S1). PhrA, a white collar-1-like blue-light photoreceptor, combines with the white collar-2 homolog phrB and binds to a specific site (5′GATA/TTG/T/AC3′) in the promoter region of the *L. edodes* tyrosinase gene [26]. The specific site (5′GATA/TTG/T/AC3′) was also searched for in the promoter regions.

### 2.3. Mycelial Materials and Environmental Treatment Methods

The parent strain W1 (ACCC50926) of W1-26 was treated as the tester strain. The strain was maintained separately on the following four mediums in dark at 25 °C: (1) malt extract, yeast extract and glucose (MYG) agar medium (2% maltose, 2% glucose, 0.2% yeast extract, 0.2% tryptone, 2% agar), (2) complete yeast extract medium (CYM) (2% glucose, 0.2% yeast extracts, 0.2% peptone, 0.1% K_2_HPO_4_, 0.05% MgSO_4_, 0.046% KH_2_PO_4_, and 2% agar), (3) CYM containing microcrystalline cellulose (CYM-C) (2% microcrystalline cellulose, 0.2% yeast extract, 0.2% peptone, 0.1% K_2_HPO_4_, 0.05% MgSO_4_, 0.046% KH_2_PO_4_, and 2% agar), and (4) CYM containing microcrystalline cellulose plus sodium lignosulfonate (CYM-C + SLS) (1.9% microcrystalline cellulose plus 0.1% sodium lignosulfonate, 0.2% yeast extract, 0.2% peptone, 0.1% K_2_HPO_4_, 0.05% MgSO_4_, 0.046% KH_2_PO_4_, and 2% agar).

**Figure 1:**
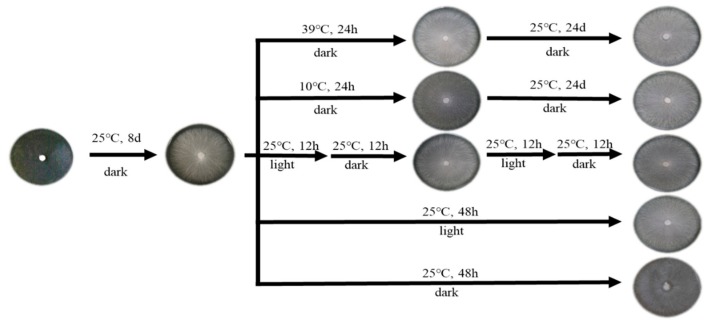
Different induction conditions for mycelia of W1 (ACCC50926).

(1) Carbon sources: W1 mycelial plugs were inoculated separately in CYM, CYM-C, and CYM-C + SLS agar medium containing 2% (wt/vol) glucose (glucose), 2% microcrystalline cellulose (cellulose), or 1.9% microcrystalline cellulose plus 0.1% sodium lignosulfonate (cellulose-SLS) as the major carbon source. Each medium was covered with cellophane and incubated for 7 days at 25 °C. Hyphae were collected, followed by freezing with liquid nitrogen and storage in a refrigerator at −70 °C for total RNA extraction. Each treatment was conducted with three biological replicates.

(2) Heat stress: A mycelial tip plug 5 mm in diameter was inoculated onto the MYG medium. For the treatment group, the mycelia grown at 25 °C for 8 days were subjected to 39 °C heat stress for 24 h, followed by transfer of the samples to incubators at 25 °C, where they were cultured for another 24 h [28] (Figure 1). Sampling was done separately at 1, 3, 6, 12, and 24 h.

(3) Cold stress: Mycelia under normal growth at 25 °C for 8 days were subjected to 10 °C cold stress for 24 h (Figure 1). The other procedures were the same as described above for heat stress.

(4) Photoperiod: A mycelial tip plug of 5 mm in diameter was inoculated onto the MYG medium and incubated for 8 days at 25 °C in the dark condition. When hyphae covered the plate, the light growth chamber was maintained at three different photoperiod regimes of 12 h light/12 h dark, 24 h light/0 h dark, or 24 h dark/0 h light at 25 °C. Sampling was done every 4 h for 48 h successively (Figure 1).

### 2.4. RNA Extraction and qRT-PCR Analysis

Total RNA from the fungus was extracted by a method adapted from the RNAiso Plus method according to the manufacturer’s instructions (TaKaRa, Dalian, China).

The transcription levels of *Lelcc1* to *Lelcc14* were analyzed using the primer sets listed in Supplementary Table S2. Additionally, white collar-1-like homolog gene *LephrA*, white collar-2 homolog gene *LephrB,* and tyrosinase homolog gene *Letry* were also analyzed. As an internal control, the expression of *actin* was analyzed using the primers Actin-F and Actin-R. The relative expression of target genes was calculated with respect to each corresponding reference condition (calibrator) (ΔΔCt = ΔCt − ΔCt _calibrator sample_). Based on the reference or a calibrator, the amount of target gene was calculated as 2^−(−ΔΔct)^ (expression) [29] and the reference condition was defined as that with the lowest expression level at the other experiments.

## 3. Results

### 3.1. Prediction and Sequence Alignment of Multicopper Oxidases in the L. edodes Genome

All 14 full-length multicopper oxidase-encoding genes were obtained in the whole genome of *L. edodes.* The detailed information regarding the laccase genes and proteins is shown in Supplementary Table S3. Except for *Lelcc3* and *Lelcc11*, all laccase proteins had signal peptide sequences, indicating that these laccases belonged to secretory proteins. The length of laccase proteins ranged from 523 aa (*Lelcc12*) to 708 aa (*Lelcc3*) residues, and the predicted molecular weights were between 56.738 kDa (*Lelcc13*) and 68.273 kDa (*Lelcc3*). The predicted pI-values of the laccase proteins ranged from 4.3 (*Lelcc5*) to 7.14 (*Lelcc11*), indicating that they belonged to acidic and neutral proteins. The intron number ranged from 9 (*Lelcc10* and *Lelcc11*) to 26 (*Lelcc6*). The coding sequences (CDS) sequences of *Lelcc1*, *Lelcc6,* and *Lelcc8* were divided into more than 20 parts by introns, indicating that these three laccases were more complicated than the other laccase genes with respect to gene structure. To determine their distribution in *L. edodes*, the *Lelcc14* genes were mapped onto *L. edodes* scaffolds, and Scaffold 0018 was identified to contain the largest number of laccase genes (*Lelcc9*, *Lelcc13*, *Lelcc14*).

**Figure 2:**
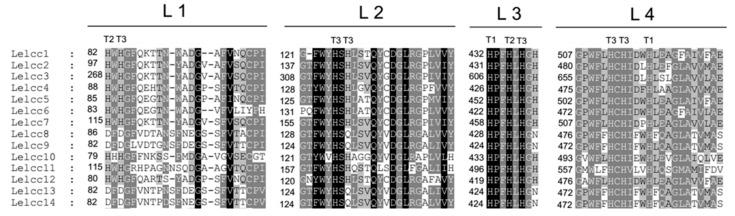
Laccase signature sequences L1–L4 and putative substrate binding loops 1–4 of laccases from *Lentinula edodes*. The histidine and cysteine residues for copper binding are indicated with T1, T2, and T3 above the residues representing type 1, type 2, and type 3 coppers, respectively.

In a previous study, 13 laccase genes, excluding *Lelcc8* in *L. edodes* D703PP-9, were analyzed and categorized into three subfamilies [11]. Phylogenetic analysis of the *L. edodes* W1-26 strain showed that *Lelcc8* could be categorized into laccase sensu stricto subfamily 2 (Supplementary Figure S1). Analysis of laccase signature sequences (L1–L4) using amino acid sequence alignment showed that all the laccases of the W1 strain had four signature sequences (Figure 2). *Lelcc8*, *Lelcc9*, *Lelcc13*, and *Lelcc14* each lacked several T2/T3 copper-binding histidines, but possessed the conserved histidines for T1 copper binding, suggesting that they were different from the other laccase genes with respect to their affinity toward T2/T3 copper ions.

### 3.2. Analysis of Stress-Related Cis-Elements in L. edodes Laccase Promoters

**Figure 3:**
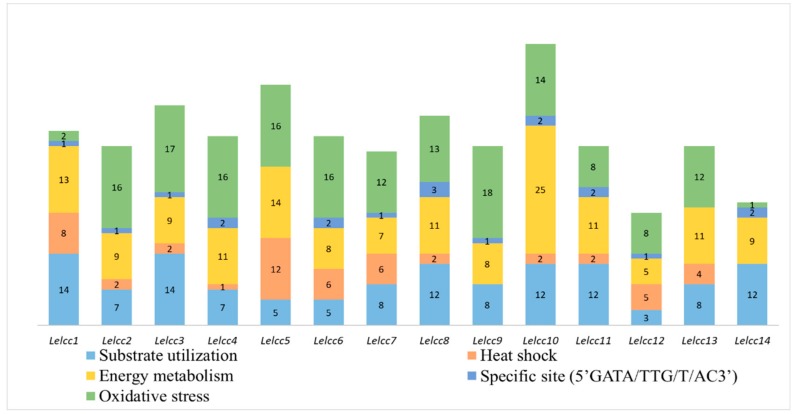
Predicted *Cis*-elements in the promoter regions of laccase genes from *L. edodes* (*Lelcc*). Promoter sequences (−1500 bp) of 14 *Lelcc* genes were analyzed, with their names shown on the bottom of the figure. *Cis*-elements with common functions are marked with the same color.

The upstream regions (1.5 kb) of the laccase sequences were obtained to search for regulation factors in response to different stress conditions. The promoter regions of *Lelcc1*–*Lelcc14* included various stress-related *Cis*-acting elements (Figure 3). Among them, *Lelcc10* had the most stress-related *Cis*-elements, including 14 oxidative stress-related, two specific sites (5’GATA/TTG/T/AC3’), 25 energy metabolism-related, two heat-shock-related, and 12 substrate utilization-related *Cis*-elements. All of the genes had *Cis*-elements related to energy metabolism, substrate utilization. and oxidative stress in the promoter regions. However, due to the limited number of stress-related *Cis*-elements in yeast types, no light and low-temperature-related *Cis*-elements were observed in the results. Almost all of the laccase genes contained *phrA*- and *phraB*-binding specific sites (5’GATA/TTG/T/AC3’) in their promoter regions. These results demonstrated that the laccase genes varied from each other in the number and type of *Cis*-acting elements, suggesting that the transcription of laccase genes in *L. edodes* could be induced by different substrates, heat stresses, photoperiods, and other stresses.

### 3.3. Expression Patterns of Lelcc genes in Three Different Carbon Sources

**Figure 4:**
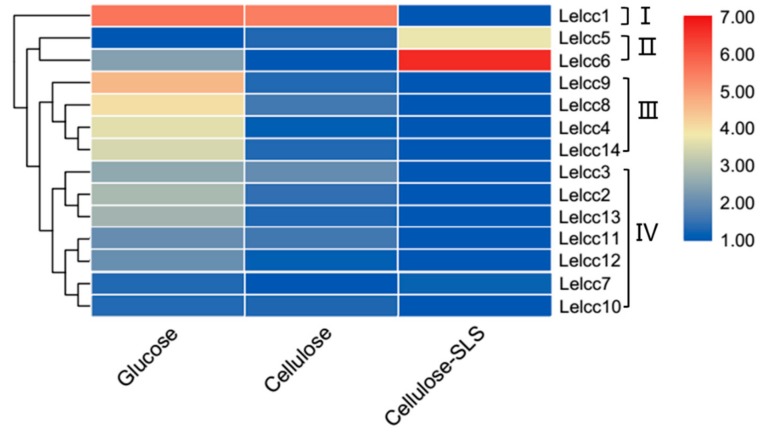
Heatmap of the expression profiles of laccase genes from *L. edodes* (*Lelcc*) in various carbon sources. The heatmaps with hierarchical clustering were visualized using the software TBtools 0.665 and the values were log_2_-transformed with normalization [30]. The blue and red elements indicate low and high relative expression levels, respectively.

Gene expression patterns are usually closely related to their functions. The expression profiles of the laccase genes were classed into four groups (I, II, III, and IV), according to their respective similar expression patterns, as shown in Figure 4 (for detail, see Supplementary Figure S2). The relative transcription levels of *Lelcc1* in CYM and CYM-C culture mediums were 48.3 and 43.9 times higher than that of the CYM-C + SLS culture medium. Compared to their expression in the other two carbon sources, the laccase genes of Group II (*Lelcc5* and *Lelcc6*) tended to be induced by the mixed carbon source (microcrystalline cellulose plus 0.1% sodium lignosulfonate), with their relative expression levels showing a 12.4- and 101.6-fold increase, respectively. These results were consistent with the comparative secretomic data published previously [27]. The most *Lelcc* genes in group III (*Lelcc4*, *Lelcc8*, *Lelcc9*, and *Lelcc14*) were observed to be transcribed almost exclusively in the CYM culture medium. The remaining seven laccases were clustered into Group IV, with higher expression levels detected in CYM or CYM-C medium than in CYM-C + SLS. Moreover, the changes in the relative expression levels of the same genes in group IV were relatively small. Collectively, the expression of most *Lelcc* genes was significantly altered in diverse carbon sources, implying that the *Lelcc* genes might be involved in fungal utilization and degradation of carbon sources.

### 3.4. Expression Patterns of Lelcc genes in Response to Heat or Cold Treatments

**Figure 5:**
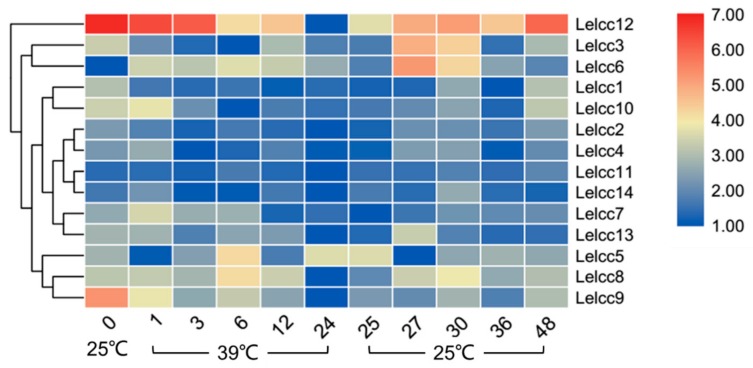
Heatmap of the expression profiles of laccase genes from *L. edodes* (*Lelcc*) under high-temperature stimulation. The heatmaps with hierarchical clustering were visualized using the software TBtools 0.665 and the values were log_2_-transformed with normalization. The blue and red elements indicate low and high relative expression levels, respectively.

As shown in Figure 5 (for detail, see Supplementary Figure S3), the relative transcription levels of most *Lelcc* genes were low at 24 h under heat stress. The relative expression of the same genes (*Lelcc1*, *Lelcc2*, *Lelcc7*, *Lelcc9*, *Lelcc10*, *Lelcc12*) showed a continuous decreasing trend after heat stress, with a 38.5- and 115.5-fold decrease in the relative expression levels of *Lelcc9* and *Lelcc12* after 24 h of heat stress. During treatment at 39 °C, the expression levels of *Lelcc7* and *Lelcc10* increased significantly at 1 h, followed by a steady decrease until 24 h. the relative expression of *Lelcc5* was significantly depressed at 1 h, upregulated until 12 h, and then upregulated again at 24 h under heat stress. The relative transcription levels of most of the *Lelcc* genes were restored after treatment at 25 °C, such as *Lelcc3*, *Lelcc6*, *Lelcc8*, *Lelcc9*, and *Lelcc12*. The laccase genes *Lelcc3*, *Lelcc6*, and *Lelcc13* presented intense changes at 25 °C after heat stress, and the peak value appeared at 27 h or 30 h. Moreover, *Lelcc3* and *Lelcc6* showed significantly higher relative expressions than most of the other laccase genes at the prophase of 25 °C recovery (27 h), followed by a gradual decrease. Collectively, high temperature had an inhibitory effect on the transcription of most laccase genes, while their relative expression levels gradually rose after returning to 25 °C.

**Figure 6:**
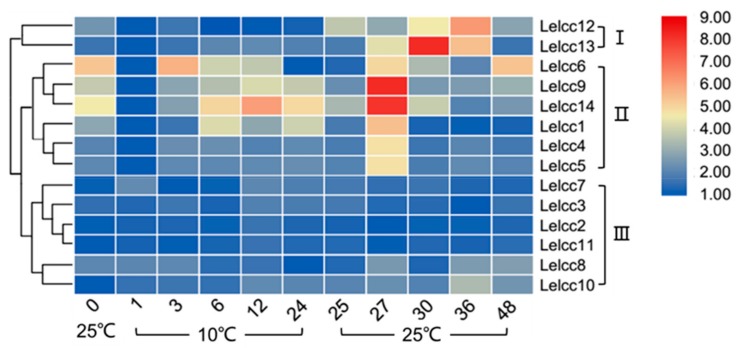
Heatmap of the expression profiles of laccase genes from *L. edodes* (*Lelcc*) under low-temperature stimulation. The heatmaps with hierarchical clustering were visualized using the software TBtools 0.665 and the values were log_2_-transformed with normalization. The blue and red elements indicate low and high relative expression levels, respectively.

In Figure 6 (for detail, see Supplementary Figure S4), the expression patterns of the *LeLcc* family were shown to vary under cold stress treatment and were divided into three groups (I, II, and III). In Group III (*Lelcc7*, *Lelcc3*, *Lelcc2*, *Lelcc11*, *Lelcc8*, *Lelcc10*), the laccase expression showed almost no striking variation at 10 °C and 25 °C, suggesting that these laccase genes were possibly not involved in the fungal response to cold stress. The transcription of genes in Group I (*Lelcc12*, *Lelcc13*) was stable most of the time during treatment at 10 °C, but was rapidly upregulated and peaked at 36 or 30 h when the temperature returned back to 25 °C, followed by a decrease. The relative transcription of *Lelcc13* showed over a 290-fold increase at 30 h. In Group II (*Lelcc6*, *Lelcc9*, *Lelcc14*, *Lelcc1*, *Lelcc4*, *Lelcc5*) the *Lelcc* genes, apart from *Lelcc6* and *Lelcc8*, exhibited a similar expression pattern to that of Group II, with a rapid upregulation and peak observed in their expression at 27 h rather than at 30 h. The relative transcription of *Lelcc9* and *Lelcc14* increased by 294- and 262-fold, respectively, compared with the lowest expression level. Overall, under 10 °C cold treatment, the transcription of some laccase genes failed to be induced, while the others varied in their expression, with most of them showing rapid up-regulation or maximal expression levels after returning to 25 °C.

### 3.5. Expression Patterns of Lelcc genes in Response to Different Photoperiods

**Figure 7:**
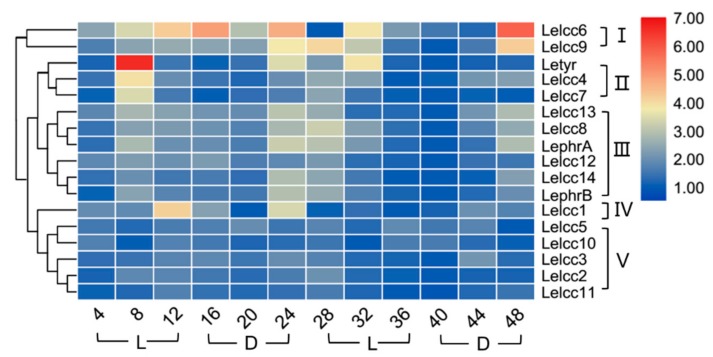
Heatmap of the expression profiles of laccase genes from *L. edodes* (*Lelcc*) in the photoperiod (12 h light/12 h dark). The heatmaps with hierarchical clustering were visualized using the software TBtools 0.665 and the values were log_2_-transformed with normalization. The blue and red elements indicate low and high relative expression levels, respectively.

Light-induced transcriptional regulation of laccase genes *LephrA*, *LephrB,* and *Letry* of *L. edodes* was analyzed by RT-qPCR using specific primers. As shown in Figure 7 and Supplementary Figure S5, the expression profiles of the *LeLcc* genes in the photoperiod of 12 h light/12 h dark were divided into five groups (I, II, III, IV, and V). Group V (*Lelcc5*, *Lelcc10*, *Lelcc3*, *Lelcc2*, *Lelcc11*) showed almost no significant variation of laccase expression, indicating that the five laccase genes may not have been involved in the light response. *Letyr*, *Lelcc4,* and *Lelcc7* were clustered to group II; their expression remained stable most of the time, but with a rapid upregulation and peak at 8 h, followed by a significant decrease at 12 h. The expression profiles of four laccase genes (*Lelcc8*, *Lelcc9*, *Lelcc13,* and *Lelcc14*) and two blue light receptor genes *(LephrA* and *LephrB*) were extremely similar, with an increase in all relative expression levels at 8 h under the light condition, followed by a sequential decrease until a peak at 24 h (in the dark), followed by another sequential decrease from dark to light, reaching the minimal level at 40 h. These results illustrated the close relationship between the four laccase genes and the two blue light receptor genes in response to light stimulus. The genes of both group II and III showed an increase in their relative expression levels at 8 h. Taken together, the laccase genes and the three known light-response genes showed extremely similar expression profiles, indicating the potential involvement of these laccase genes in the molecular response of *L. edodes* to light.

**Figure 8:**
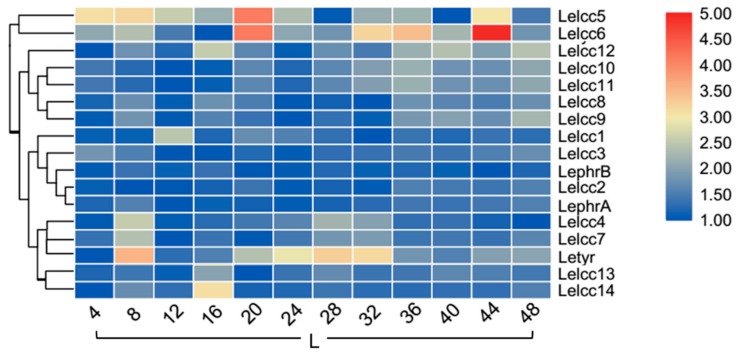
Heatmap of the expression profiles of laccase genes from *L. edodes* (*Lelcc*) in the photoperiod (24 h light/0 h dark). The heat maps with hierarchical clustering were visualized using the software TBtools 0.665 and the values were log_2_-transformed with normalization. The blue and red elements indicate low and high relative expression levels, respectively.

**Figure 9:**
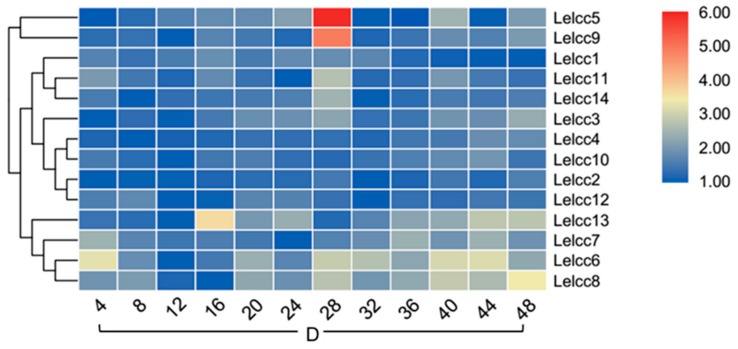
Heatmap of the expression profiles of laccase genes from *L. edodes* (*Lelcc*) in the photoperiod (0 h light/24 h dark). The heatmaps with hierarchical clustering were visualized using the software TBtools 0.665 and the values were log_2_-transformed with normalization. The blue and red elements indicate low and high relative expression levels, respectively.

To further understand how light regulates the expression of laccase genes, the expression patterns of laccase genes under continuous illumination (24 h light/0 h dark) and continuous darkness (0 h light/24 h dark) were examined (Figure 8, Supplementary Figure S6, Figure 9, and Supplementary Figure S7). Intuitively, the relative expression levels showed a smaller variation in the photoperiod of 24 h light/0 h dark and the photoperiod of 0 h light/24 h dark than in the photoperiod of 12 h light/12 h dark. In the photoperiod of 24 h light/0 h dark, *LephrA* and *LephrB* showed no significant fluctuation in their relative expression levels. The two laccase genes (*Lelcc4* and *Lelcc7*) and the tyrosine gene (*Letyr*) were still clustered to the same group, with their relative expressions peaking at 8 h. In the continuous dark photoperiod of 0 h light/24 h dark, with increasing time, the expression showed little difference in *Lelcc4* and *Lelcc7*. This result indicated that the expression of *Lelcc4* and *Lelcc7* may have been induced by light and was closely related to tyrosinase.

## 4. Discussion

In 2015, 13 of the 14 laccases, excluding *Lelcc8,* were identified in *L. edodes* D703PP-9 [11]. In this study, we analyzed all 14 *laccase* genes in *L. edodes* W1-26. The majority of the fungal laccases are extracellular monomeric globular proteins of approximately 50–70 kDa, with an acidic isoelectric point (pI) around pH 4.0 [7]. The characteristic analysis results of the proteins in this report were similar to those of previous studies, indicating that the laccases of *L. edodes* are the typical ones. Most of the laccase secreted to the outside of the cell is acidic [31]. The fungal laccase amino acid sequence generally contains a signal peptide sequence at the N-terminus to guide transmembrane transfer [17]. In this study, except for LELCC3 and LELCC11, the laccases possessed the signal peptide, while in *L. eodes* D703PP-9, LELCC3 contained the signal peptide. Additionally, the L1 sequences of LELCC8, LELCC9, LELCC13, and LELCC14 in *L. edodes* W1-26 are more complete than those in *L. edodes* D703PP-9 [11]. This indicates that the laccase genes not only vary between different species, but also between different varieties. Signal peptide sequence deficiency was also reported in *Flammulina velutipes* and *Setosphaeria turcica* [32,33], as well as in plants [34], implying that the laccase gene may have lost the signal peptide sequence during evolution.

The original laccase gene is differentiated into paralogous genes with different functions to fulfill the various functional requirements of fungi throughout the life cycle [35]. Previous studies showed that it was common for fungal laccase genes to be clustered on the same scaffold. In *P. ostreatus*, 12 *PoLac* genes were mapped to six scaffolds and in *L. edodes* strain D703PP-9, 13 laccase genes were mapped to seven scaffolds [17,36,37]. In the current research, the 14 laccase genes were unevenly scattered among eleven scaffolds, probably due to the imperfect genome assembly of the reference genome in W1-26.

In this research, many stress-related *Cis*-elements were observed in the nucleotide sequences extending 1500 bp upstream of the *Lelccs* and were classified into five major groups, such as oxidative stress-related, specific sites (5′GATA/TTG/T/AC3′), energy metabolism-related, heat-shock-related, and substrate utilization-related *Cis*-elements. *Cis*-elements were shown to play significant roles in the regulatory process of responding to multiple abiotic stresses [38]. These results implied that *Lelcc* genes could be involved in fungal response processes toward multiple stresses. In heterokaryons, different nuclear types exist within a single cell of *L. edodes*. In this research, the parent dikaryotic strain W1 was used to detect gene expression levels, while the genome data of W1-26, a single spore culture derived from W1, were used for the *Cis*-elements analysis. This may be responsible for the inconsistency between the gene expression patterns and the prediction of *Cis*-elements. Widespread transcriptomic variation was identified between the nuclear types of *Agaricus bisporus* and *L. edodes*, and two different nuclei contributed differently to the regulation of the fungal cells [39,40]. The relationship between the promoter *Cis*-elements and gene expression levels of *Lelccs* should be studied further.

Understanding the mechanism of lignocellulose degradation by *L. edodes* is critical. The expression pattern was detected by qRT-PCR for the laccase family members in *L. edodes,* which was cultured with three different carbon sources and divided into four groups by clustering analysis. The relative transcription levels of *Lelcc1*, *Lelcc5,* and *Lelcc6* were consistent with our previous comparative secretome analysis in different carbon sources (microcrystalline cellulose, lignosulfonate, and glucose) [27]. This indicates the reliability of the qRT-PCR analysis used to detect the relative transcription levels of the genes in this study. In this study, the relative transcription levels of group II (*Lelcc5* and *Lelcc6*) were induced by lignosulfonate, highlighting their roles in ligocellulose degradation, which was consistent with a previous study reporting that the laccases were involved in lignin degradation [41]. Conversely, group III (the last 12 laccase genes) was observed almost exclusively in glucose culture. Previous studies showed that rich available glucose in the medium led to cellular oxidative stress, and laccase of *L. edodes* can be assumed to have a protective role rather than a direct role in lignocellulose deconstruction [27]. Oxidative stress was observed to stimulate the extracellular laccase activity of some white rot basidiomycetes, such as *Fomes fomentarius*, *Tyromyces pubescens*, *Trametes versicolor*, and *Abortiporus biennis* [42,43]. The transcriptome and exoproteome analysis of *Dichomitus squalens* demonstrated that laccase adapted well to the changing substrate [44]. Similar results were reported in the white-rot fungi *Pycnoporus coccineus* and *Phlebiopsis gigantea* [45,46]. Overall, the expression profile of laccase genes varied significantly in diverse groups, implying that the genes in the fungal laccase family may have functionally differentiated during adaptation to the changing environment, and the process of carbon source utilization requires high coordination among multiple laccases. This assumption needs to be further elucidated in future studies.

Proper cold stimulation is essential to induce the differentiation of hyphae from primordia to fruiting bodies [47]. In the present study, the expression profile of the *Lelcc* gene was analyzed during different temperature treatments. At the onset of the low-temperature induction, the expressions of some laccase genes (Group I and II) were significantly reduced, probably due to the rapid response of laccases at low temperatures. This was similar to a report regarding laccase activities in *Pleurotus tuoliensis* [12]. The relative expressions of most laccase genes reached the highest level during 25 °C recovery phases. These results were consistent with the findings regarding *P. tuoliensis* [12]. *L. edodes* is a variable temperature fruiting mushroom and the subsequent process of fruiting-body development may require a large amount of carbohydrates. Laccases are involved in the degradation of lignocellulose into available sugar for fungi during development [12]. Thus, a large quantity of laccases could be induced to degrade lignin and cellulose for fruiting-body development after low-temperature induction. Overexpression of *lcc1* in *H. marmoreus* resulted in the induction of primordium initiation about 3–5 days earlier in the transgenic fungus [18]. To date, the regulation of laccase expression and the role of mushroom laccase during adaptation to temperature change has not been thoroughly elucidated.

Heat stress significantly inhibits the growth of mycelium and the formation of fruit bodies [47]. In the heat treatment, the relative expression of most laccase genes showed a continuous decreasing trend when the mycelia of *L. edodes* were grown at 39 °C, in contrast to an increasing trend at 25 °C after heat stress. The mycelial activity and growth rate of *L. edodes* decreased in the presence of high temperatures [28,48,49]. Thus, high temperature inhibits the transcription of the laccase genes, which may be responsible for mycelial activity.

In this study, the expression profiles of *Lelccs* (*Lelcc13*, *Lelcc14*) were extremely similar to those of *LephrA* and *LephrB*. Two genes with similar expression profiles were reported to be regulated by the same transcription factor in *Populus* [50]. This result indicated that *Lelcc13*, *Lelcc14,* and blue-light photoreceptors were regulated by the same transcription factor or had similar functions. The transition from the vegetative stage in darkness to the reproductive stage in light may have resulted in sudden metabolic changes, such as carbon and tyrosine metabolism involved in melanin synthesis [22,51]. The expression patterns of laccases in group II (*Lelcc4*, *Lelcc7*) were clustered into the same group as tyrosinase of *L. edodes*. Laccase is the third enzyme with a tyrosinase domain, other than tyrosinase and polyphenol oxidase (PPO); these three enzymes are extremely important for melanin production [52]. The pigment production induced by light in brown film formation stage was identified as melanin (unpublished). *Lelcc4* and *Lelcc7* may be involved in the browning of mycelium by effective melanin synthesis and light induction, a theory which is supported by previous reports showing increased transcription of laccase during the browning of mycelium under light treatment [22,51] and the involvement of *lcc4* from *L. edodes* in melanin synthesis [53].

## 5. Conclusions

In summary, this study demonstrated the comprehensive expression profile of 14 laccase genes from *L. edodes* under different environmental stresses. The relative expression levels of the 14 genes were significantly influenced by various growth conditions, indicating the involvement of *Lelcc*s in during adaptation to the changing environment. Additionally, the result of the dendrogram implied the potential existence of functional divergence among the 14 laccase genes of *L. edodes* in response to different stresses. This study provided valuable information contributing toward the understanding of the adaptive capacity of white-rot fungi to the environment and laccase engineering research.

## Supplementary Materials

The following are available online at https://www.mdpi.com/2073-4425/10/12/1045/s1, Figure S1: Phylogenetic analysis of laccases in *Lentinula edodes*, Figure S2: Expression profiles of *Lelcc* genes in various carbon sources. Results are presented as mean ± standard deviation (SD), Figure S3: Expression profiles of *Lelcc* genes in heat stress, Figure S4: Expression profiles of *Lelcc* genes in low-temperature stress, Figure S5: Expression profiles of *Lelcc* genes in photoperiod (12h light/12h dark), Figure S6: Expression profiles of *Lelcc* genes in photoperiod (24h light/0h dark), Figure S7: Expression profiles of *Lelcc* genes in photoperiod (0h light/24h dark), Table S1: Transcription factor binding sites predicted by Yeastract, Table S2: Primers used in this study. Table S3: *Lelcc* members identified in *L. edodes*.
