# Supplementary material for: Expression Profile of Laccase Gene Family in White-Rot Basidiomycete Lentinula edodes under Different Environmental Stresses
Source: Genes (Basel). 2019 Dec 16;10(12):1045. doi: 10.3390/genes10121045 (PMC6947313; doi:10.3390/genes10121045)
Supplement: Supplementary file 1 [file genes-10-01045-s001.pdf]

Table 1. factor binding sites predicted by Yeastract.

Classification	Element Name	Signal Sequence	Putative Function
Substrate utilization	Adr1p	TTGGRG	Carbon source-responsive zinc-finger transcription factor
	Azf1p	AAGAAAAA	Zinc-finger transcription factor, involved in induction of CLN3 transcription in response to glucose
	Cat8p	NCCDTYNVNCCGN	active after diauxic shift, binds carbon source responsive elements
	Cst6p	GTGACGT	Basic leucine zipper (bZIP) transcription factor of the ATF/CREB family involved in utilization of non-optimal carbon sources and chromosome stability
	Gat1p	GATAAG	Transcriptional activator of genes involved in nitrogen catabolite repression;
	Gzf3p	GATAAG	GATA zinc finger protein and Dal80p homolog that negatively regulates nitrogen catabolic gene expression; function requires a repressive carbon source
	Nrg1p	CCCTC	Transcriptional repressor that recruits the Cyc8p-Tup1p complex to promoters; mediates glucose repression
	Rgt1p	CGGANNA	Glucose-responsive transcription factor that regulates expression of several glucose transporter (HXT) genes in response to glucose
	Sip4p	NCCDTYNVNCCGN	C6 zinc cluster transcriptional activator that binds to the carbon source-responsive element (CSRE) of gluconeogenic genes;
Heat shock	Hsf1p	NGAANNTTCN	Trimeric heat shock transcription factor, activates multiple genes in response to stresses that include hyperthermia
	Skn7p	GGCYGGC	required for optimal induction of heat-shock genes in response to oxidative stress; involved in osmoregulation
Energy metabolism	Gcr1p	CTTCC	Transcriptional activator of genes involved in glycolysis; DNA-binding protein that interacts and functions with the transcriptional activator Gcr2p
	Gsm1p	CGGNNNNNNNNNCGG	Putative zinc cluster protein of unknown function; proposed to be involved in the regulation of energy metabolism, based on patterns of expression and sequence analysis
Oxidative stress	Stb5p	CGGNS	Transcription factor, involved in regulating multidrug resistance and oxidative stress response; forms a heterodimer with Pdr1p
	Yap1p	TGACTCA	Basic leucine zipper (bZIP) transcription factor required for oxidative stress tolerance

Table 2. used in this study.

Primer	Sequence (5′to 3′)	Description
lcc1-QF<br>lcc1-QR	GCAGGGTAGTCGCTATCGTTTT<br>TTATCAGCAGTTTGATTTGCAGTG	Detects the *Lelcc1* gene expression
lcc2-QF<br>lcc2-QR	CAGGCACCTTCTGGTATCATTCA<br>TCGGCAATAGTAAGCACGGTAGT	Detects the *Lelcc2* gene expression
lcc3-QF<br>lcc3-QR	TCCCTGTTCTACAATCGGCACT<br>GTGTACGGATTCGGCGAGTAA	Detects the *Lelcc3* gene expression
lcc4-QF<br>lcc4-QR	TCAGGCTGGCAAGCGTTATAG<br>TGATTAGCGGTCAAAACAAAGGA	Detects the *Lelcc4* gene expression
lcc5-QF<br>lcc5-QR	ACGACGACACCACCATTATC<br>CAGCCAAATCAGTGGCATTAC	Detects the *Lelcc5* gene expression
lcc6-QF<br>lcc6-QR	TACCCTCACTGGGACCGTATGT<br>CGAAAGAGTGATGACCGTGGA	Detects the *Lelcc6* gene expression
lcc7-QF<br>lcc7-QR	TAGTATCCACTGGCACGGTCTTT<br>AGCCGTCGCAGTATTGAACC	Detects the *Lelcc7* gene expression
lcc8-QF<br>lcc8-QR	TGGTATCGTCCCAGTTTCGG<br>AGTCATTGGAGTGGTCGGAGTT	Detects the *Lelcc8* gene expression
lcc9-QF<br>lcc9-QR	GCATCCCTTCCACCTCCAT<br>CGGGCACAACTCAGTCCAG	Detects the *Lelcc9* gene expression
lcc10-QF<br>lcc10-QR	TTGGTTTCAACGAGAACTCTACGC<br>AATCGGATATTCTTCGGTGTCTGTA	Detects the *Lelcc10* gene expression
lcc11-QF<br>lcc11-QR	ACAGTGCTTACGGTCAATGGTCT<br>GTGCCAATGTAAACTGGTAGCCT	Detects the *Lelcc11* gene expression
lcc12-QF<br>lcc12-QR	TGGATTGCGTGGAGCCTTTG<br>TCGGGACAGTACCGCTTTGG	Detects the *Lelcc12* gene expression
lcc13-QF<br>lcc13-QR	ATCCGACCAGGAACTTGAACCA<br>GCAGTAACCACGAGATTGAGGG	Detects the *Lelcc13* gene expression
lcc14-QF<br>lcc14-QR	AACACTGAGAATCCCATAAAGCG<br>GCCATCACAGTAGCGAGACCAG	Detects the *Lelcc14* gene expression
phrA-F<br>phrA-R	CACTTCCTCGACTGGAATTAGG<br>GTACTGCCAACTCGATTCTCTAC	Detects the *LephrA* gene expression
phrB-F<br>phrB-R	CTGAAGACGGGCATAAGAAGAA<br>GTAGTCCACAAGCGTTACAGAG	Detects the *LephrA* gene expression
tyr1-F<br>tyr1-R	TGGTTTGTCCATTCGGTAGAGC<br>AGTGGATGATAAGAGGGGTAGGC	Detects the *Letyr1* gene expression
Actin-F<br>Actin-R	GGAGAAGATTTGGCATCACACA<br>GAAGAGCGAAACCCTCGTAGA	Detects the *Leactin* gene expression

Table 3. *gene* (*Lelcc*) members identified in the genome of *L. edodes* monokaryotic strain W1-26.

Gene name	Locus name	Location	Introns	Group	SP	Size(aa)	MW(Da)	PI
Lelcc1	LE01Gene08330	LE01Scaffold0049:34167-39465	24	1-A	Y	537	57526.37	4.57
Lelcc2	LE01Gene07443	LE01Scaffold0040:103186-106086	13	1-B	Y	533	57880.26	4.94
Lelcc3	LE01Gene07056	LE01Scaffold0036:220857-224325	15	1-B	N	708	78630.47	6.76
Lelcc4	LE01Gene04008	LE01Scaffold0014:408733-412091	16	1-B	Y	527	57099.02	5.37
Lelcc5	LE01Gene01372	LE01Scaffold0004:121357-125473	18	1-A	Y	554	59531.51	4.3
Lelcc6	LE01Gene13044	LE01Scaffold0127:1869-5175	26	1-A	Y	532	56891.49	5.36
Lelcc7	LE01Gene07758	LE01Scaffold0043:97003-100271	12	1-B	Y	560	61029.93	5.69
Lelcc8	LE01Gene01361	LE01Scaffold0004:91720-95445	23	2	Y	528	57097.05	4.56
Lelcc9	LE01Gene04648	LE01Scaffold0018:221426-227240	15	2	Y	524	56798.59	4.32
Lelcc10	LE01Gene05032	LE01Scaffold0020:315527-318125	9	F	Y	631	68273.61	4.6
Lelcc11	LE01Gene06108	LE01Scaffold0028:269081-271435	9	F	N	597	66893.97	7.14
Lelcc12	LE01Gene08149	LE01Scaffold0047:72666-75950	19	2	Y	523	57242.03	4.66
Lelcc13	LE01Gene04737	LE01Scaffold0018:454217-456795	15	2	Y	524	56738.83	4.75
Lelcc14	LE01Gene04660	LE01Scaffold0018:260417-264015	14	2	Y	524	56760.71	4.46

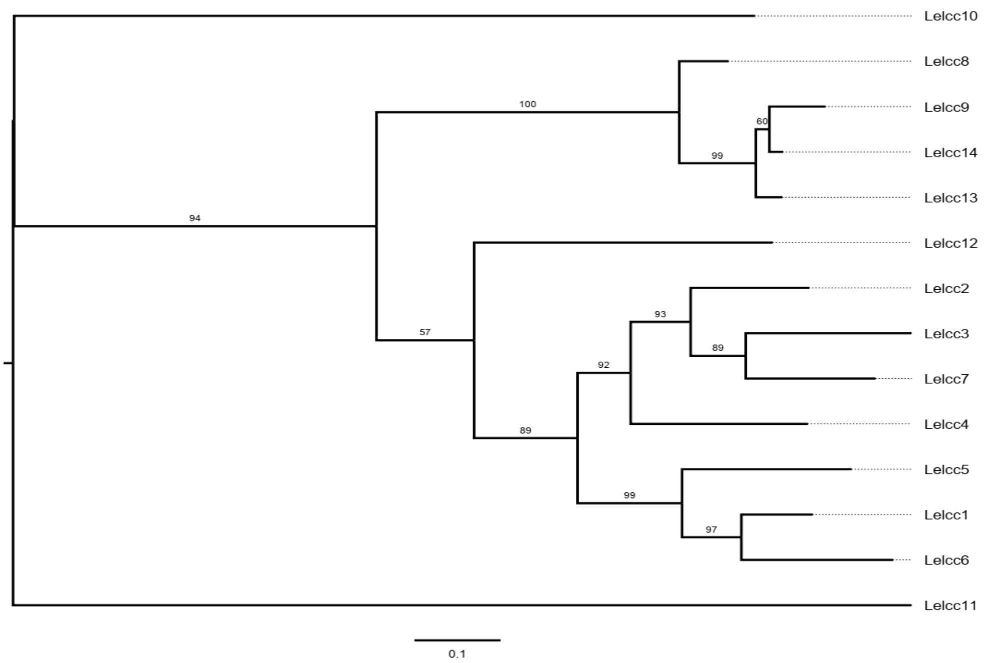

Figure 1. Phylogenetic analysis of laccases in *Lentinula edodes (Lelcc)*. The phylogram was constructed using the neighbor-joining method. The scale bar indicates a distance of 0.07, and the numbers on branches indicate percentage bootstrap support values (based on 1000 replications).

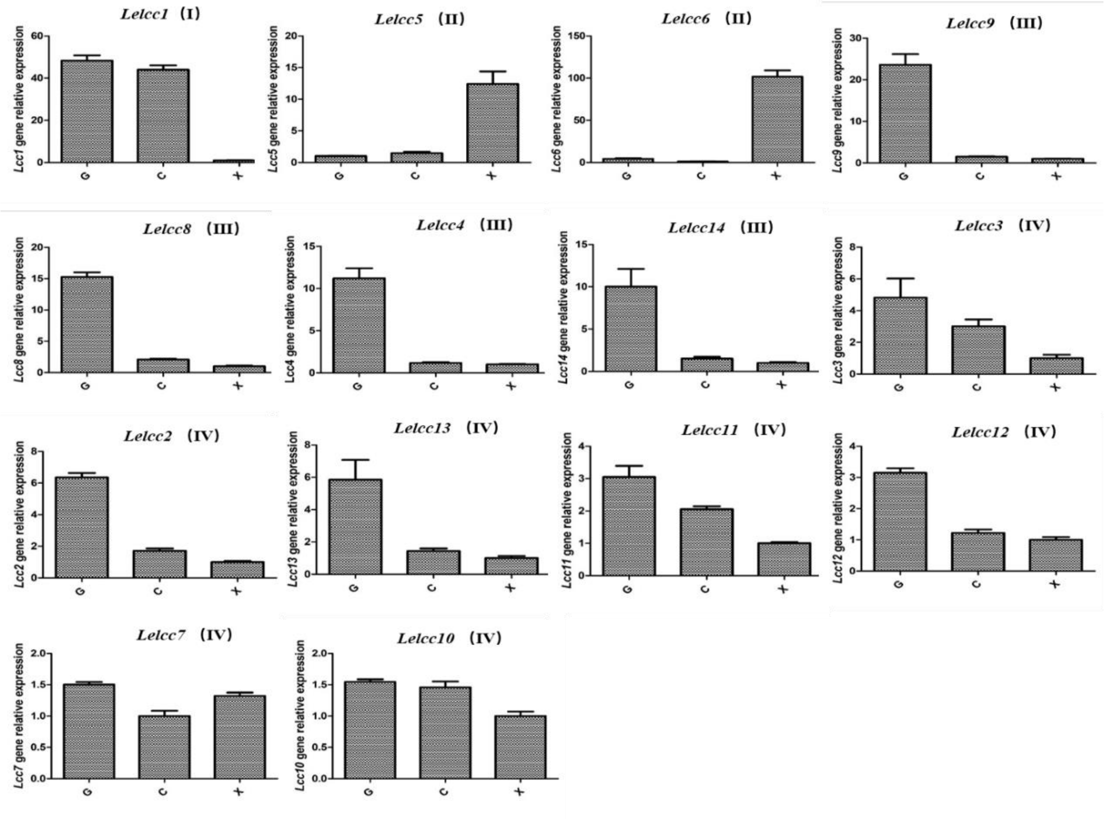

Figure 2. Expression profiles of laccase genes from *L. edodes* (*Lelcc*) in various carbon sources. Results are presented as mean ± standard deviation (SD).

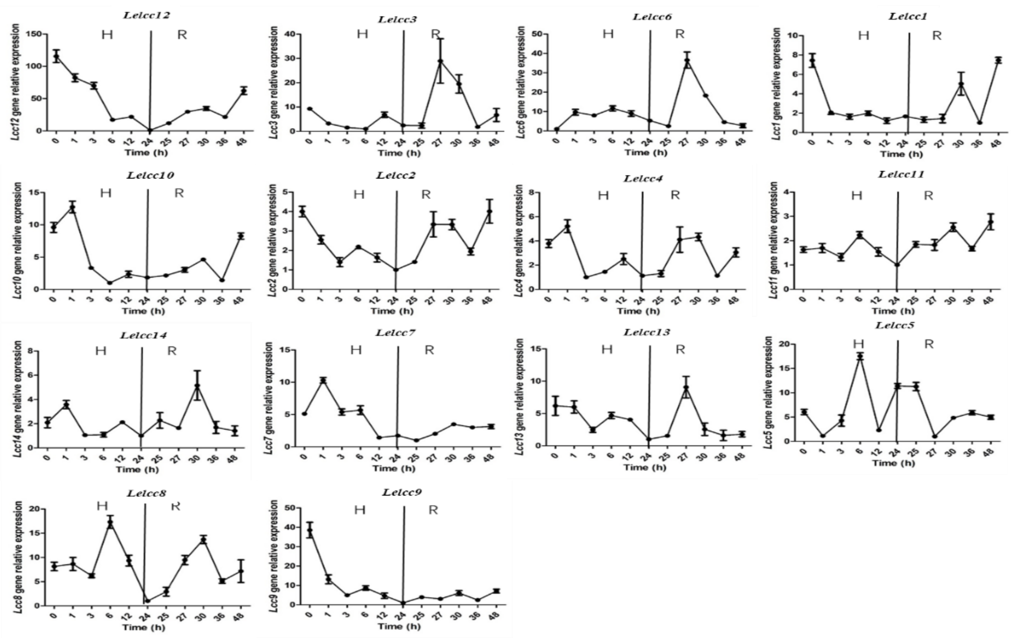

Figure 3. Expression profiles of laccase genes from *L. edodes* (*Lelcc*) in heat stress. H: 39°C high temperature stress treatment phase; R: 25°C recovery phase. Results are presented as mean ± standard deviation (SD).

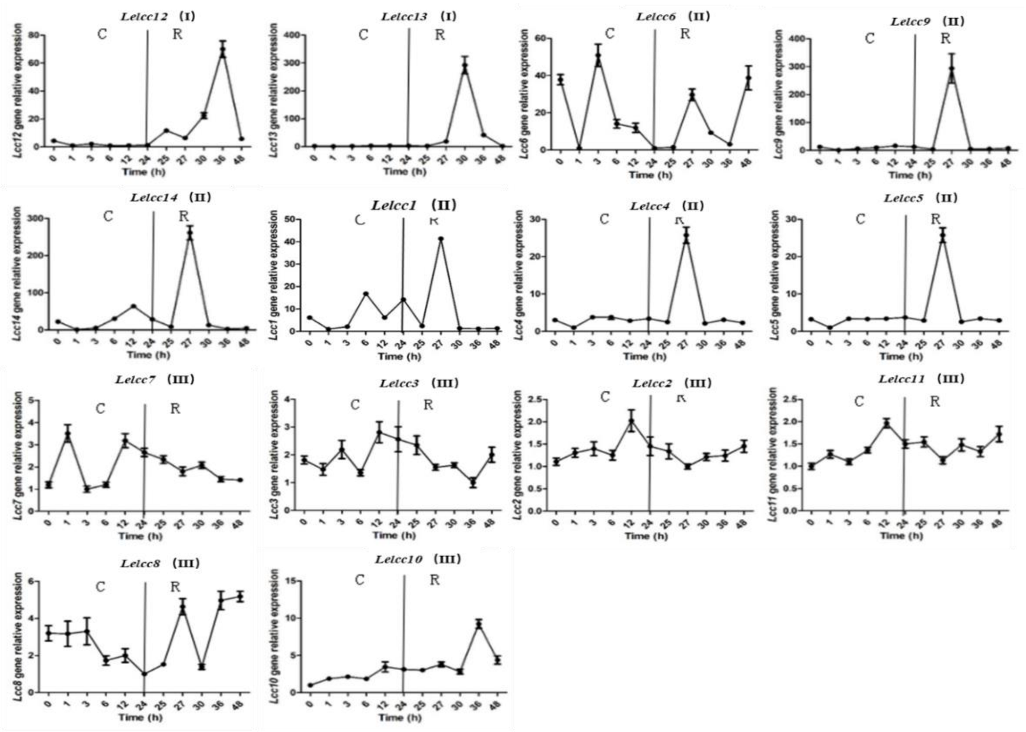

Figure 4. Expression profiles of laccase genes from *L. edodes* (*Lelcc*) in low-temperature stress. C: 10°C low-temperature stress treatment phase; R: 25°C recovery phase. Results are presented as mean ± standard deviation (SD).

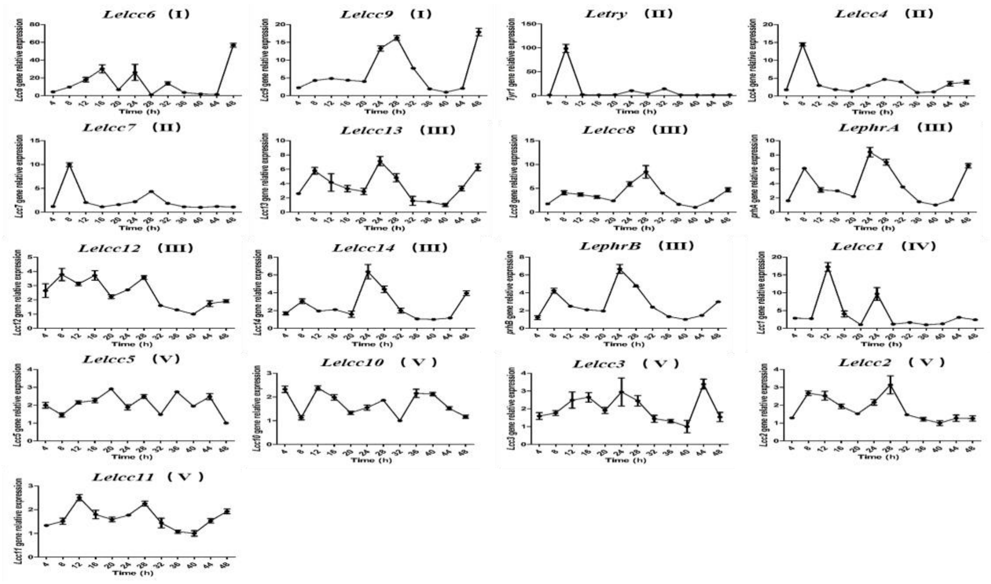

Figure 5. Expression profiles of laccase genes from *L. edodes* (*Lelcc*) genes in photoperiod (12h light/12h dark). L: light; D: dark. Results are presented as mean ± standard deviation (SD).

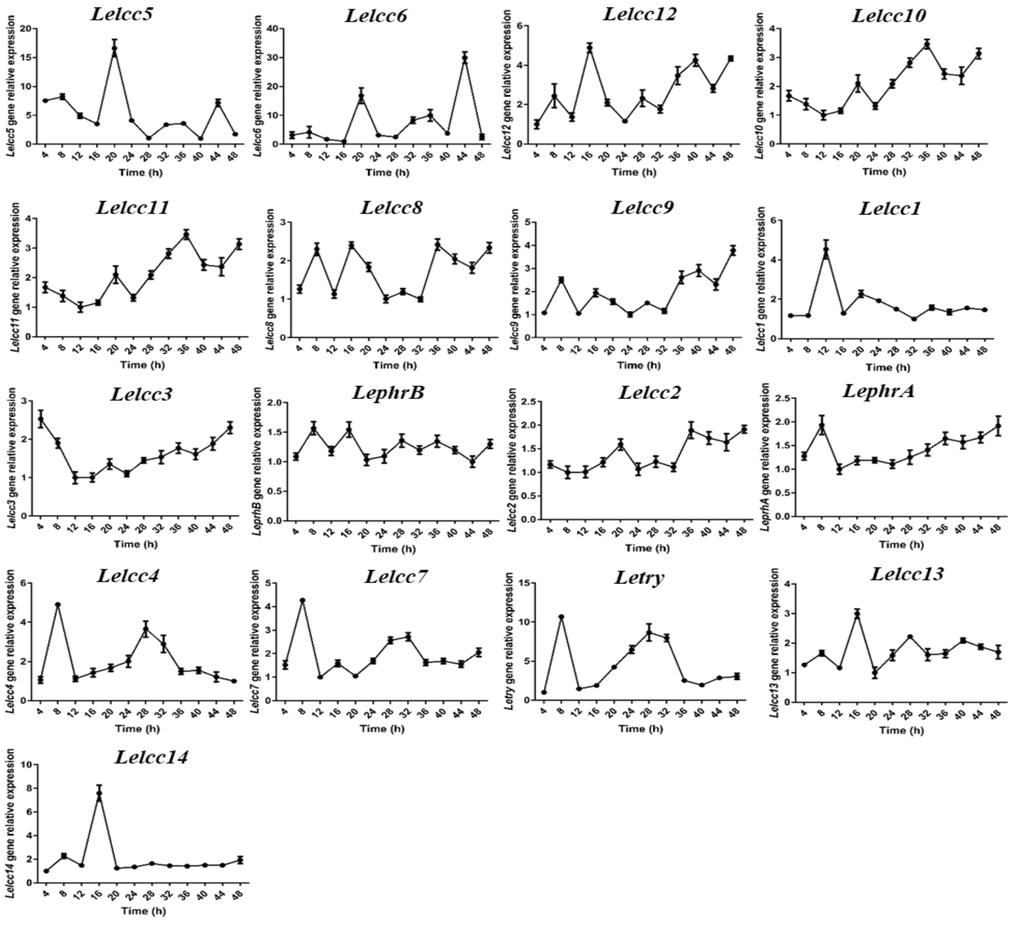

Figure 6. Expression profiles of laccase genes from *L. edodes* (*Lelcc*) in photoperiod (24h light/0h dark). Results are presented as mean ± standard deviation (SD).

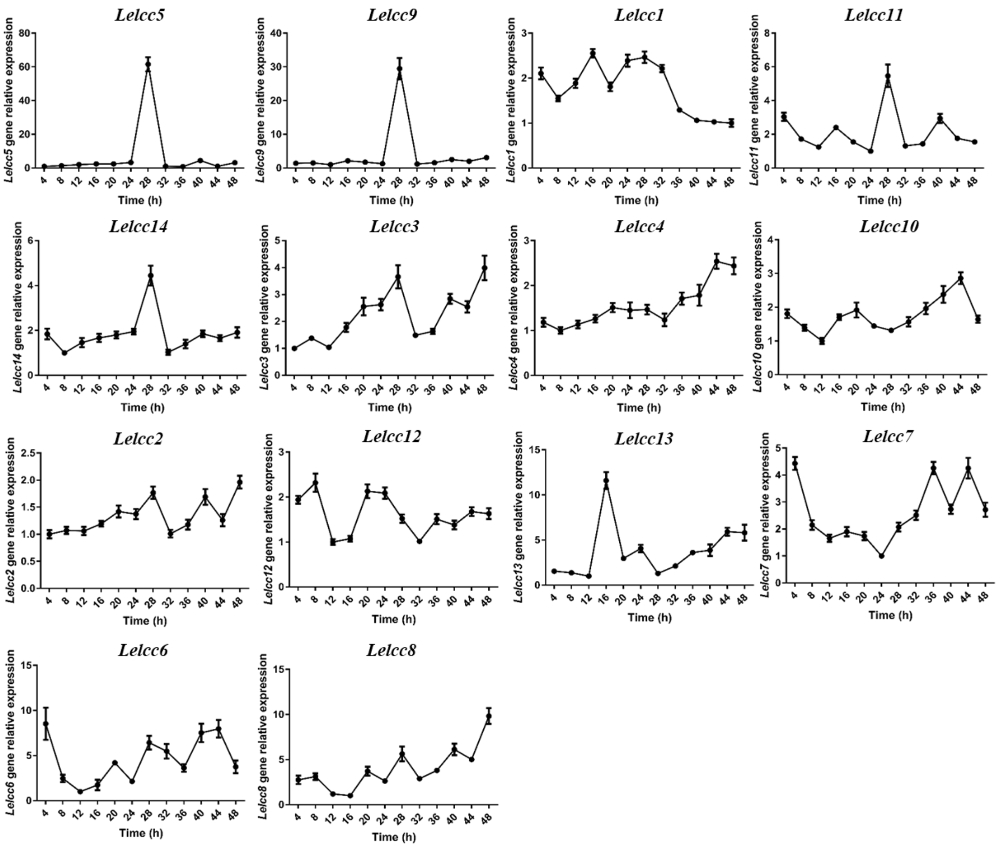

Figure 7. Expression profiles of laccase genes from *L. edodes* (*Lelcc*) in photoperiod (0h light/24h dark). Results are presented as mean ± standard deviation (SD).
